# Supplementary material for: Content-specific activity in frontoparietal and default-mode networks during prior-guided visual perception
Source: eLife. 2018 Jul 31;7:e36068. doi: 10.7554/eLife.36068 (PMC6067880; doi:10.7554/eLife.36068)
Supplement: Transparent reporting form [file elife-36068-transrepform.docx]

***eLife’s* transparent reporting form**

We encourage authors to provide detailed information *within their submission* to facilitate the interpretation and replication of experiments. Authors can upload supporting documentation to indicate the use of appropriate reporting guidelines for health-related research (see [EQUATOR Network](http://www.equator-network.org/%20)), life science research (see the [BioSharing Information Resource](https://biosharing.org/)), or the [ARRIVE guidelines](http://www.plosbiology.org/article/info:doi/10.1371/journal.pbio.1000412) for reporting work involving animal research. Where applicable, authors should refer to any relevant reporting standards documents in this form.

If you have any questions, please consult our Journal Policies and/or contact us: [editorial@elifesciences.org](mailto:editorial@elifesciences.org).

**Sample-size estimation**

- You should state whether an appropriate sample size was computed when the study was being designed
- You should state the statistical method of sample size computation and any required assumptions
- If no explicit power analysis was used, you should describe how you decided what sample (replicate) size (number) to use

Please outline where this information can be found within the submission (e.g., sections or figure legends), or explain why this information doesn’t apply to your submission:

Information about sample-size estimation can be found in the *METHODS: Subjects* section. The sample size was chosen based on previous 7T fMRI studies on perceptual / cognitive functions (*Emmerling et al., Neuroimage 2016; Sengupta et al., Neuroimage 2017; Suthana et al., J Cogn Neurosci 2015; De Martino et al., Nat Comm 2012; Klein et al., Neuron 2014; Yang et al., Neuroimage 2014*). Our sample size (n = 23) was considerably larger than these previous studies (range 5-15). After excluding subjects with excessive movements in the scanner, the final sample size was n = 19.

**Replicates**

- You should report how often each experiment was performed
- You should include a definition of biological versus technical replication
- The data obtained should be provided and sufficient information should be provided to indicate the number of independent biological and/or technical replicates
- If you encountered any outliers, you should describe how these were handled
- Criteria for exclusion/inclusion of data should be clearly stated
- High-throughput sequence data should be uploaded before submission, with a private link for reviewers provided (these are available from both GEO and ArrayExpress)

Please outline where this information can be found within the submission (e.g., sections or figure legends), or explain why this information doesn’t apply to your submission:

Information about replicates and data exclusion can be found in the *METHODS: Subjects* section. Our experiment was performed in n = 23 human participants, which constitutes a biological replicate. Four subjects were excluded due to excessive movement in the scanner.

**Statistical reporting**

- Statistical analysis methods should be described and justified
- Raw data should be presented in figures whenever informative to do so (typically when N per group is less than 10)
- For each experiment, you should identify the statistical tests used, exact values of N, definitions of center, methods of multiple test correction, and dispersion and precision measures (e.g., mean, median, SD, SEM, confidence intervals; and, for the major substantive results, a measure of effect size (e.g., Pearson's r, Cohen's d)
- Report exact p-values wherever possible alongside the summary statistics and 95% confidence intervals. These should be reported for all key questions and not only when the p-value is less than 0.05.

Please outline where this information can be found within the submission (e.g., sections or figure legends), or explain why this information doesn’t apply to your submission:

Details about statistical analyses are included in the METHODS section as well as in the corresponding RESULTS section and figure legends, when pertinent.

Most of the analysis were performed at the population level, correcting for multiple comparisons across voxels or ROIs. For massive-univariate analysis, we employed the FLAME1 estimation method to perform cluster-wise inference (see *METHODS: GLM Analysis*). To correct for multiple comparisons, a cluster-defining threshold of p < 0.001 (z > 3.1) and minimum cluster size of 17 voxels was applied (corresponding to p < 0.05, FWE-corrected). As shown in Fig. 1 of Eklund et al. (PNAS 2016), the family wise error rate of FLAME1 method (with a cluster-defining threshold of z > 3.1) applied to an event-related design is very much in the acceptable range.

For MVPA searchlight analysis, a non-parametric permutation-based approach was used to assess significance, with 5000 shuffles. To perform cluster-wise inference, a threshold-free cluster enhancement algorithm (Smith & Nichols, 2009) was then used (p < 0.01, FWE-corrected).

For ROI-based analyses, false discovery rate (FDR) correction across ROIs was used. Given the large number of ROIs (20), we often report whether an effect is significant under a fixed cut-off (i.e. p < 0.05, FDR-corrected). However, we report the exact p-value in cases of marginally or not significant results.

We do not report effect size estimates (such as Cohen’s *d*) for decoding and RSA results, since they are not informative for these analyses (see *Hebart & Baker, Neuroimage 2017, Deconstructing decoding for the study of brain function*). However, we report goodness-of-fit measures (r^2^ and stress) for multidimensional scaling (MDS) solutions obtained from RSA results (see *METHODS: RSA* section).

(For large datasets, or papers with a very large number of statistical tests, you may upload a single table file with tests, Ns, etc., with reference to sections in the manuscript.)

**Group allocation**

- Indicate how samples were allocated into experimental groups (in the case of clinical studies, please specify allocation to treatment method); if randomization was used, please also state if restricted randomization was applied
- Indicate if masking was used during group allocation, data collection and/or data analysis

Please outline where this information can be found within the submission (e.g., sections or figure legends), or explain why this information doesn’t apply to your submission:

This information can be found in the *METHODS: Task paradigm* section. Samples (participants) were randomly allocated to the only group of the study. All participants were exposed to all combinations of our experimental factors. The order of Mooney image presentation was randomized across subjects.

**Additional data files (“source data”)**

- We encourage you to upload relevant additional data files, such as numerical data that are represented as a graph in a figure, or as a summary table
- Where provided, these should be in the most useful format, and they can be uploaded as “Source data” files linked to a main figure or table
- Include model definition files including the full list of parameters used
- Include code used for data analysis (e.g., R, MatLab)
- Avoid stating that data files are “available upon request”

Please indicate the figures or tables for which source data files have been provided:

Interactive 3-dimensional MDS plots corresponding to first-order RDMs for each ROI can be found at: <https://gonzalezgarcia.github.io/mds.html> . Corresponding 2-dimensional MDS plots are shown in Fig. 3C and Fig. 4B.

As source data, we provide individual-level representational dissimilarity matrices (RDMs) for all ROIs (Fig. 3-6) and individual-level representational similarity matrices (RSMs) based on behavioral performance for all ROIs (Fig. 7). Additionally, we provide MATLAB code to reproduce first-order RSA (Figs. 3 & 4), second-order RSA (Fig. 5), dimensionality (Fig. 6), and preservation index (Fig. 7) analyses.

Source data for individual-level behavioral results (Fig. 1) and decoding accuracy maps (Fig. 2) are also available, but exceed the data file size limit on the submission website. These data are available upon request.
